# Enhancement of blood-brain barrier permeability by the combination of bubble liposomes and high-intensity focused ultrasound

**DOI:** 10.1186/2050-5736-3-S1-P30

**Published:** 2015-06-30

**Authors:** Yoichi Negishi, Masaya Yamane, Naho Kurihara, Yoko Endo-Takahashi, Norio Takagi, Ryo Suzuki, Kazuo Maruyama, Yukihiko Aramaki

**Affiliations:** 1 Tokyo University of Pharmacy and Life Sciences, Hachioji, Japan; 2 Teikyo University, Itabashi-ku, Japan

## Background/introduction

The blood-brain barrier is a major obstacle that prevents therapeutic drugs or genes being delivered into the central nervous systems. Therefore, it is important to develop enabling enhance the permeability of blood-brain barrier (BBB). So far, we have developed echo-contrast gas (C3F8) entrapping liposomes (Bubble liposomes, BLs) which can be work as a gene delivery tool in the combination of ultrasound (US) exposure. Here, we investigated whether the delivery efficiency of intravenously injected large molecular agents can be induced by the combination of BLs and high-intensity focused ultrasound (HIFU).

## Methods

In this study, we prepared BLs of submicron size (~ 500 nm). Male ICR mice were injected intravenously with Evans blue dye (EB) which binds with albumin in blood stably. Subsequently, BLs were also injected and then right hemispheres were exposed with a 1.0 MHz pulsed HIFU (10% duty, 10-60 sec) with different intensities (0.1-1.5 kW/cm^2^). After 3 hours, their treated mice were infused intravenously with PBS as a perfusion medium by a syringe pump at a constant speed. The mice were perfused with PBS via the left ventricle. After perfusion and brain removal, the brains were then divided into right and left hemispheres before measuring the amount of EB extravasated. Non-focused left hemispheres of the treated mice were used as the control. Samples were weighed, soaked in formamide solution, and the incubated for 24 hours at 55 degrees. After that, the extracted dye was finally determined using a spectrophotometer at 620 nm. Similarly, FITC-dextrans (molecular weight 70-2000 kDa) were also delivered intravenously into right hemispheres by the combination of BLs and HIFU. Then, sections of the treated brains were examined by fluorescence microscopy.

## Results and conclusions

The accumulations of EB in the focused on brain were increased in dependent on the intensity and the duration of HIFU. In contrast, non-focused left hemispheres of the treated mice were almost background level. Furthermore, it was also shown that delivery in the efficient brain of a compound (molecular weight 70-2000 kDa) becomes possible by the combination method of Bubble liposome and HIFU. These results suggest that BBB permeability after the treatment of BLs and HIFU can be enhanced. It was thought that the method of combining Bubble liposome and HIFU together served as a useful means as the technique of accelerating the permeability of BBB. Therefore, it may be expected as a future low invasive drugs or genes delivery system within a brain.

